# Knowledge, Awareness and Vaccination Attitude Towards HPV in Sex and Gender Minorities: A Cross-Sectional Study

**DOI:** 10.3390/vaccines13050508

**Published:** 2025-05-12

**Authors:** Antonio Di Lorenzo, Paola Berardi, Andrea Martinelli, Francesco Paolo Bianchi, Giovanni Migliore, Silvio Tafuri, Pasquale Stefanizzi

**Affiliations:** 1 Dipartimento Interdisciplinare di Medicina, Università Degli Studi di Bari Aldo Moro, 70121 Bari, Italy; antoniodilorenzo95@gmail.com (A.D.L.); berardipaola28@gmail.com (P.B.); silvio.tafuri@uniba.it (S.T.); 2 Public Health Department, ASL Brindisi, 72100 Brindisi, Italyfrapabi@gmail.com (F.P.B.); 3 Apulia Agency for Health and Social Policies, 70121 Bari, Italy; gmfiaso@gmail.com

**Keywords:** questionnaire, LGBTQ+, vaccine hesitancy, SGMs

## Abstract

**Background/Objectives**: Sex and gender minorities (SGMs) include individuals who do not comply with sexual binarism and heteronormative standards. They represent a high-risk population for Human Papillomavirus (HPV) infection and potential target of an HPV vaccine offer. This study investigates SGMs’ knowledge, awareness and vaccination attitude regarding HPV. **Methods**: This is a cross-sectional, questionnaire-based study. The target population was represented by SGMs living in Italy and using social media platforms of SGM rights associations. The study questionnaire was based on the literature and disseminated via said associations’ social media. It included items regarding knowledge and awareness, expressed as seven-point Likert scales, and questions about personal information, sexual anamnesis and vaccination attitude. Data collection started on 1 November 2023 and ended on 8 December 2023. **Results**: The questionnaire was answered by 177 people. Knowledge and awareness scores were generally high (45.98 ± 6.14 and 34.21 ± 4.62, respectively). Regarding attitude, 31.64% of participants reported being hesitant or refusing HPV vaccination, mainly due to prohibitive costs or low perception of the vaccine’s utility. Higher education was associated with a higher knowledge score (coeff.: 2.25; 95%CI: 0.69–3.82); likewise, a history of HPV-related lesions positively influenced the score (coeff.: 2.47; 95%CI: 0.20–4.75). The awareness score was only increased by a greater number of sexual partners (coeff.: 0.06; 95%CI: 0.01–0.11). Older age was proven to significantly increase the odd of vaccine hesitancy (OR: 1.07; 95%CI: 1.02–1.12). **Conclusions**: Despite a good level of knowledge and awareness, the study population manifested significant barriers to vaccination. The main ones were related to the vaccine’s cost and lack of medical information. Future efforts should focus on reinforcing vaccine offers to SGMs.

## 1. Introduction

Human Papillomavirus (HPV) is the most common sexually transmitted pathogen [1,2,3], with an estimated prevalence of 11.7% worldwide [4]. Due to its high prevalence and contagiousness, HPV is currently thought to infect over 90% of sexually active men and 80% of sexually active women at least once during their life [3]. Some HPV genotypes may infect their host persistently and determine cancer of various body districts, including cervical, penile, anal, vaginal, vulvar and head and neck cancer [3,4].

To date, three anti-HPV vaccines are available: a bivalent product (2vHPVv) targeting the high-risk genotypes HPV-16 and 18, a quadrivalent product (4vHPVv) for the high-risk genotypes HPV-16 and 18 as well as low-risk HPV-6 and 11, and a nine-valent vaccine (9vHPVv) providing protection against the high-risk genotypes HPV-16, 18, 31, 33, 45, 52 and 58 and against the low-risk genotypes HPV-6 and 11 [5,6,7,8]. All products are based on inactivated viral sub-units, which spontaneously arrange themselves in virus-like particles [6,7,8]. While 2vHPVv is only used in females, 4vHPVv and 9vHPVv have been approved for use in both male and female subjects aged between 9 and 26 [9].

In Italy, two doses of 9vHPVv are offered actively and free-of-charge to all adolescents at ages 11–12. All females aged 11–26 and males aged 11–18 can access vaccination for free, with a three-dose schedule being used for subjects over 15 [10]. Since the vaccine is highly immunogenic (99–100% seroconversion rate) and protective antibodies persistence covers most of the sexual experimentation phase (77.5–100% 5-year persistence), no booster doses are required [5].

Sex and gender minorities (SGMs) include all individuals who do not comply with sexual binarism and heteronormative standards due to their own sexual orientation, anatomical characteristics, gender identity and/or gender expression [11]. SGMs have a higher risk of developing oncological diseases, generally stemming from behavioural risk factors and often inconsistent sexually transmitted disease (STD) screening [12,13], and HPV-related cancers also have higher incidence among these subjects [14]. The HPV infection rate is also significant in SGMs and is usually associated with high-risk sexual behaviours, a high number of sexual partners and inconsistent or inadequate use of barrier contraceptives [15,16].

Female-to-male transexuals (trans men, TM), moreover, represent a hard-to-reach population due to their anatomical, psychological and social profile. Indeed, only a minority of these subjects undergo gender reassignment hysterectomy [17]. However, cervical cancer screening might be difficult to offer: firstly, these patients might change their sexual identity in online registries after transitioning, thus making it difficult to identify them as screening targets; secondly, even when contacted, TM often refuse the very idea of “having a uterus”, since the sole presence of this organ is perceived as distressing [18].

Despite all this, the literature regarding knowledge and risk awareness about HPV in SGMs is scarce, as well as evidence regarding SGMs’ attitude towards HPV vaccination. Our paper investigates these aspects within the Italian SGM population. Our aim is to highlight the liabilities of current vaccination and communication strategies in order to propose possible interventions.

## 2. Materials and Methods

This was a cross-sectional, questionnaire-based observational study. Our target population was represented by people belonging to SGMs residing in Italy, including cisgender homosexual and bisexual men and women, non-binary people and transgender men and women. No age limits were set for inclusion. Only cisgender, heterosexual men and female were excluded.

The questionnaire was based on the existing literature [9,11,12,19,20]. Validation was carried out via the Cronbach’s alpha test [21], employing a randomly selected twenty-person group for testing. The questionnaire’s final version was disseminated online via social media channels of the LGBT rights associations MiXED lgbtqia+ and Arcigay; the former is a regional organization, while the latter is the main national association for LGBT rights in Italy. Data collection started on 1 November 2023 and ended on 8 December 2023.

The questionnaire (see Appendix A) was made of 40 items, divided into four sections:

1. Personal information (in anonymous form) and sexual anamnesis.
2. HPV-related knowledge (8 questions).
3. HPV infection risk awareness (6 questions).
4. Attitude towards HPV vaccination.

In Section 2 and Section 3, questions were formulated as 1-to-7 Likert scales, in which 1 meant “I fully disagree with this statement” and 7 meant “I fully agree with this statement”. An English-translated copy of the questionnaire is available in Appendix A. All questions required answering to proceed; this design choice was made in order to avoid interviewees skipping items.

The questionnaire was made via the online application Google Form^®^. The database was built via Microsoft Excel^®^ (v.16.90.2). All statistical analyses were performed via StataMP17^®^. Written information was provided in the questionnaire’s form, and explicit consent was requested for data collection and usage. No ethical committee approval was required, since data were provided anonymously and voluntarily, and no personal information was collected. All data were managed in accordance with the European General Data Protection Regulation [22]. This research was carried out in compliance with the Declaration of Helsinki and within the scope of public-health-oriented activities of the Apulian Regional Epidemiology Observatory.

### Statistical Analysis

Since this study was designed for specific web platforms’ users, the addressed population approximated to the followers of social media profiles to whom the questionnaire was disseminated. The official Arcigay association’s Instagram^®^ page dedicated to health topics (@healthypeers) was chosen, since it had the largest user base. In fact, despite not forming the entirety of its user base, SGMs are the page’s main target. Followers amounted to circa 2900 people on 1 November 2023; therefore, a minimum sample size of 112 individuals was calculated, considering a type I error of 5% on both tails and a type II error of 30% [23].

Quantitative variables were defined as mean ± standard deviation. Qualitative variables were defined as percentage (proportion). To each Likert scale answer, a correctness score was given. The score corresponded to the interviewee’s answer for correct statements, while it amounted to 8 (answer) for incorrect statements. A general score was then calculated for the knowledge and awareness sections, ranging from 8 to 56 for the former and 6 to 42 for the latter.

The sections’ mean scores were correlated with the participants’ age and sexual partner number via Pearson’s correlation test. Mean scores were also correlated among subjects based on their education level, gender identity, history of Human Immunodeficiency Virus (HIV) infection or HPV-related lesions, transgender status and disclosure of gender identity/sexual orientation to a healthcare provider. Quantitative variables were tested for normality, but distribution was found to be non-normal; non-parametrical testing (Kruskal–Wallis’s test, Mann–Whitney’s test) was therefore employed for correlation.

Vaccination attitude was expressed as the presence/absence of vaccine hesitancy or refusal, identified via the question “Do you intend to get vaccinated against HPV?”, whose possible answers were “Yes”, “No” and “I already am”. It was correlated among the aforementioned groups via the chi-squared test. The correlation between vaccine hesitancy and the subject’s age and number of sexual partners was then studied via the Pearson correlation test.

Knowledge score (KS) and awareness score (AS) and vaccination attitude were finally studied via association tests. Regarding KS and AS, for each of them, a multivariable linear regression model was built; for vaccination attitude, a multivariable logistic regression model was fitted. In all cases, the independent variables were the following: age (years), gender identity (male, female, non-binary), transgender status, education level (both as education degree and years in education), number of reported sexual partners in the last 12 months, disclosure to a healthcare provider, occurrence of STD screening recommendation by a healthcare provider, history of HIV infection and history of HPV-related lesions. Independent variables which showed a significant association with our main outcomes were then studied individually via univariable regression models, either linear or logistic.

Due to the exploratory nature of this study, no correction methods were employed for type I error. This choice was taken in order to avoid excessive conservativity and based on the existing literature, considering that the risk of type I error inflation was less relevant than the risk of excessive conservativity [24,25,26]. For all statistical tests, a two-sided *p*-value < 0.05 was considered as an indicator of statistical significance.

## 3. Results

### 3.1. Descriptive Statistics

During the dissemination period, 177 participants were recruited. Their average age was 30.14 ± 7.87 years. The sample composition in terms of gender identity was 39.55% (70/177) women, 50.85% (90/177) men and 9.60% (17/177) non-binary people.

Among the interviewees, 9.04% (16/177) identified as transgender people, and nine of these subjects declared that they were undergoing or had undergone transition. No-one reported undergoing gender reaffirmation hysterectomy. Regarding education level, 5.08% (9/177) of participants only achieved a middle school degree or lower. An additional 35.03% (62/177) reported graduating from high school, while 59.89% (106/177) declared they were in possession of higher education degrees. The mean reported time in education amounted to 15.74 ± 2.96 years.

Having had one or more sexual partners with male external genitals during the last 12 months was reported by 66.10% (117/177) of participants, with a mean of 8.32 ± 15.66 partners. Having had sexual partners with female external genitals was reported by 29.38% (52/117) of participants, with a mean of 1.71 ± 1.23 partners. Finally, 16.38% (29/177) of interviewees declared they had no sexual partners over the last 12 months. Overall, 6.00 ± 13.24 sexual partners were reported by our study population during the previous 12 months, and 51.41% (91/177) of subjects stated that they had a stable sexual partner for all or part of the same period.

Regular STD screening was reported by 43.50% (77/177) of participants. A screening recommendation by a healthcare provider was reported by 13.56% (24/177) of interviewees. Furthermore, 35.03% (62/177) of subjects had disclosed their SGM status to one or more healthcare providers. A history of HIV infection was reported by 4.52% (8/177) of participants, while 19.77% (35/177) of them reported a history of HPV-related lesions. A total of 97.74% (173/177) of the sample declared they had at least heard of HPV, while 88.70% (157/177) knew a vaccine existed for HPV.

**Figure 1:**
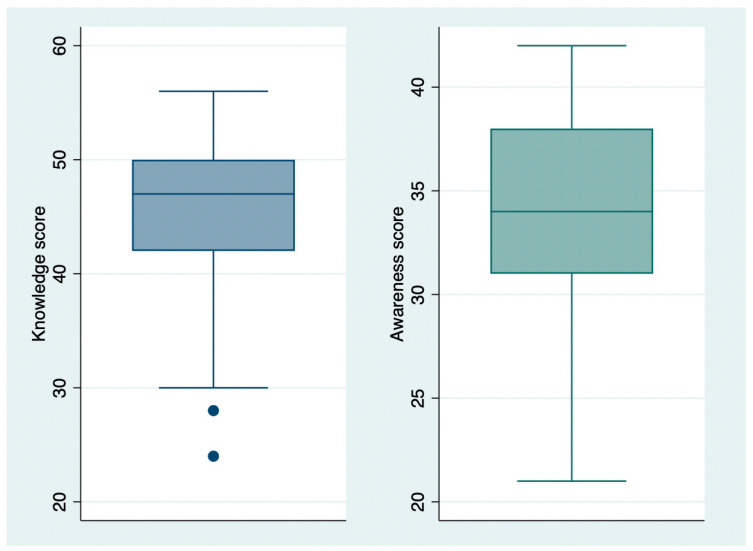
Knowledge (blue) and awareness (green) score distribution.

**Figure 2:**
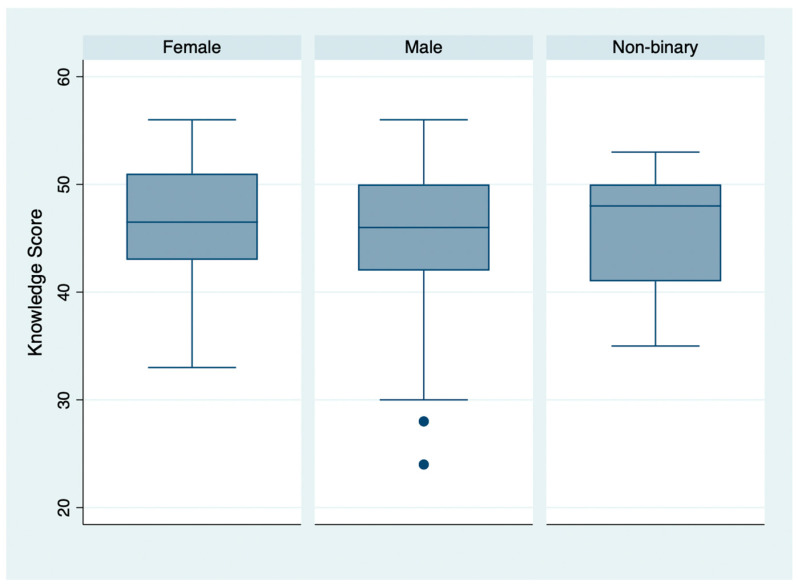
Knowledge score distribution, by gender identity.

**Figure 3:**
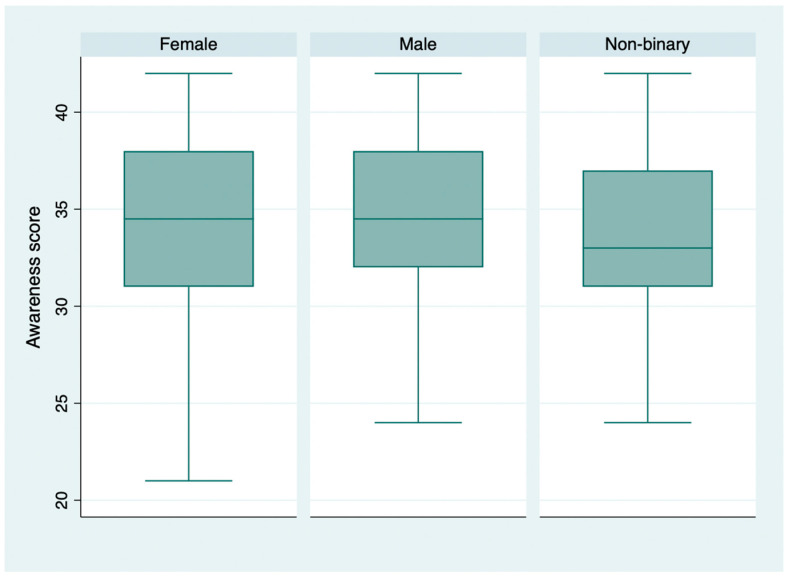
Awareness score distribution, by gender identity.

The mean KS was 45.98 ± 6.14, while the mean AS was 34.21 ± 4.62 (Figure 1, Figure 2 and Figure 3). The individual questions’ scores are reported in Table 1. Regarding vaccination attitude, 31.64% (56/177) of participants were hesitant or refused HPV vaccination, while 44.63% (79/177) reported already being vaccinated, and 23.73% (42/177) stated intention to vaccinate.

The most commonly reported reasons for vaccine hesitancy were the following: perceived unnecessity or uselessness of vaccination (10.73%, 19/177), excessive cost of vaccination (9.60%, 17/177) and lack of knowledge regarding a vaccine’s existence (8.47%, 15/177). Only 2.83% (5/177) of participants reported a fear of adverse events as their reason for being hesitant. When asked “If you do not intend to vaccinate, what would change your mind?”, hesitant interviewees stated that information by medical professionals, an active call system dedicated to SGMs and explicit recommendations for all SGMs would represent facilitators for vaccine acceptance.

Information about the study population is summarized in Table 2.

### 3.2. Inferential Statistics

#### 3.2.1. Knowledge

Pearson’s test highlighted a significant direct correlation between KS and age (r: 0.17; *p*-value < 0.05). The same method did not show a significant correlation between KS and the number of sexual partners (r: 0.04; *p*-value > 0.05).

A significant difference was identified by the Kruskal–Wallis’s test in the mean KS of subjects with different levels of education (chi2: 8.06; *p*-value: 0.018); this result was confirmed by Pearson’s correlation test when school years were considered, with a significant direct correlation with the KS (r: 0.25; *p*-value < 0.05). Subjects with different gender identities, on the other hand, did not show significant differences in terms of the mean KS score (chi2: 0.39; *p*-value: 0.821).

Mann–Whitney’s test did not highlight significant KS differences between transgender and cisgender subjects (z: 1.02; *p*-value: 0.305). In a similar fashion, no significant difference was observed in KS between subjects who had disclosed their SGM status to a healthcare provider and those who had not (z: 1.09; *p*-value: 0.276).

Regarding clinical history of STDs, subjects with a history of HIV infection did not show significantly different knowledge than those with no diagnosis of infection (z: 0.86; *p*-value: 0.392). A significant difference was instead highlighted between subjects with and without a history of HPV-related lesions (z: −2.31; *p*-value: 0.021).

The fitted multivariable regression model showed a significant direct association between a subject’s education and their KS (coeff.: 2.25; 95%CI: 0.69–3.82; *p*-value: 0.005). A history of HPV-related lesions was also significantly associated with a higher KS (coeff.: 2.47; 95%CI: 0.20–4.75; *p*-value: 0.033). All other variables did not show significant influence on the score (*p*-value > 0.05).

Results were confirmed by univariable linear regression for both education level (coeff.: 2.58; 95%CI: 1.09–4.08; *p*-value: 0.001) and history of HPV-related lesions (coeff.: 2.76; 95%CI: 0.51–5.02; *p*-value: 0.017).

#### 3.2.2. Awareness

Pearson’s correlation test was not significant for AS and age (r: 0.11; *p*-value > 0.05). However, the number of sexual partners reported for the last 12 months was significantly correlated with this score (r: 0.21; *p*-value < 0.05).

No significant AS differences were observed between subjects with different instruction levels (chi2: 2.174; *p*-value: 0.337), and Pearson’s test confirmed this observation when school years were considered (r: 0.13; *p*-value > 0.05). Likewise, the mean AS did not differ significantly in subjects with different gender identities (chi2: 1.03; *p*-value: 0.596).

The mean AS was not significantly different between transgender and cisgender subjects when studied via the Mann–Whitney test (z: 1.09; *p*-value: 0.275). The mean score did not change significantly according to SGM status disclosure, either (z: −0.69; *p*-value: 0.492).

People with a previous diagnosis of HIV seropositivity did not show significantly different ASs when confronted with subjects with no such history (z: −1.18; *p*-value: 0.238). Moreover, subjects with and without a history of HPV-related lesions did not present significant differences in AS (z: −1.18; *p*-value: 0.075).

Multivariable linear regression identified a significant influence on AS only as far as sexual partner number is concerned (coeff.: 0.06; 95%CI: 0.01–0.11; *p*-value: 0.034). All other independent variables were not proven to significantly impact this score (*p*-value > 0.05). Univariable regression confirmed the significant effect of sexual partners’ numerosity (coeff.: 0.07; 95%CI: 0.02–0.12; *p*-value: 0.005).

#### 3.2.3. Vaccine Hesitancy

Pearson’s test highlighted a significant direct correlation between vaccine hesitancy and the participants’ age (r: 0.18; *p*-value < 0.05). The same test did not show a significant correlation between vaccine hesitancy and last year’s sexual partner number (r: −0.14; *p*-value > 0.05).

The chi-squared test did not show significant differences in terms of vaccine hesitancy when gender identity (chi2: 0.89; *p*-value: 0.640), transgender status (chi2: 0.36; *p*-value: 0.549), education level (chi2: 1.92; *p*-value: 0.383), SGM status disclosure to a healthcare provider (chi2: 0.78; *p*-value: 0.376), HIV seropositivity (chi2: 0.17; *p*-value: 0.679) or history of HPV-related lesions (chi2: 2.73; *p*-value: 0.098) was taken into consideration.

A multivariable logistic regression model highlighted significantly higher odds of vaccine hesitancy for older subjects (OR: 1.07; 95%CI: 1.02–1.12; *p*-value: 0.005). However, no other variable was proven to significantly influence the risk of vaccine hesitancy (*p*-value > 0.05). The significant effect of older age was confirmed by univariable logistic regression (OR: 1.05; 95%CI: 1.01–1.09; *p*-value: 0.022).

## 4. Discussion

Our study population showed a good level of knowledge regarding HPV infection and the diseases it causes. Likewise, interviewees were generally well aware of their risk of becoming infected. However, significant gaps were identified in some aspects, particularly regarding extra-genital localization of HPV-related cancers (mean score: 4.56 ± 2.32). Moreover, despite a generally high risk awareness, many participants reported not perceiving this risk as relevant to themselves (mean score: 4.66 ± 2.36).

Our results are only partially coherent with those obtained by other studies. The recent literature confirms, in fact, that people belonging to SGMs tend to have a good level of knowledge about HPV but also reports gaps and misinformation related to the uncertainty about target populations for HPV vaccination and the actual risk of HPV-related malignancies [27]. On the other hand, several studies found knowledge of HPV and its vaccine in SGMs wanting and confirmed this lack of knowledge in the general population [28,29,30,31]. It should be noted, though, that these studies only include men who have sex with men (MSM), excluding women, transgender and non-binary people.

Our study’s approach to the target population was also different: while the aforementioned studies generally relied on paper-based questionnaires administered in either sexual health centres dedicated to subjects with a known history of STDs, or informal venues dedicated to sexually active MSM with no stable partners, our investigation employed an online questionnaire. Further, the questionnaire was disseminated via the official social media profiles of organizations whose main purpose is communication towards SGMs. In addition to this, all the cited studies reported data prior to 2019, and therefore our results might have benefitted from national and international health authorities’ communication efforts over the last five years [32,33].

As far as vaccination attitude is concerned, our study highlighted a significant hesitancy level, with 31.64% of participants reporting indecision or straight-up refusal of HPV vaccination. On the other hand, 44.63% of the sample population declared that they had already been vaccinated, and an additional 23.73% reported intention to vaccinate. These figures are in line with the SGM-related literature, which shows suboptimal vaccine coverage but a positive attitude towards vaccination in these population groups [27,28,34,35].

The reasons for hesitancy reported by our population also find confirmation in the existing literature. The cost of HPV vaccination is a well-known barrier, especially for those who are not targeted by vaccination campaigns due to age and therefore have to face expenses [35,36,37,38,39]. Complacency, or a perceived uselessness of vaccination, is also one of the main determinants of vaccine hesitancy [40]. This factor, in SGMs, is enhanced by sporadic offers of HPV vaccination by healthcare providers [36,41,42,43,44,45].

The associations highlighted by our regression models are generally unsurprising. The greater knowledge about HPV in subjects with higher education may be related to a greater degree of health literacy and information. People with a history of HPV-related lesions, on the other hand, may have acquired a more detailed knowledge regarding their own condition thanks to the healthcare providers who cared for them [36,46].

Interestingly, participants with a higher number of sexual partners showed significantly higher risk awareness. This contradicts the existing literature [47,48] and might be related to the greater circulation of information regarding HPV in the last few years. The channels we chose for the questionnaire’s dissemination may have also influenced our results: they often offer educational contents dedicated to SGMs, and our study population may therefore present a bias due to the attendance of this virtual environment.

Our study population’s attitude towards vaccination was influenced by the subjects’ age; the odds of hesitancy, in fact, were greater in older people. Other studies have already described this phenomenon [49,50], which might be related to either a lower perceived usefulness of vaccination in subjects who have already initiated sexual activities or a greater stability of sexual life in mature adults rather than adolescents and young adults. However, most studies regarding HPV vaccine hesitancy are oriented to parents of minors eligible for vaccination, rather than minors themselves [51,52]; data, therefore, are scarce.

To our knowledge, this is the first cross-sectional study investigating knowledge, awareness and vaccination attitude about HPV in people belonging to SGMs, not MSM alone. The questionnaire we used was based on the literature and on the best knowledge regarding HPV and vaccine hesitancy dynamics. We also took into consideration a large number of confounders, in order to minimize their impact on final results.

A few weaknesses must be addressed. First of all, the low sample number surely influenced the potency of this study, especially regarding less represented groups such as non-binary persons, and would therefore allow for sub-analyses. However, we designed this investigation as a pilot study with the potential to guide further research. For the same reason, questions regarding structural barriers to vaccination, ethnicity and socio-economic factors were not included in the item set but should be included in future research.

Secondly, the questionnaire only targeted SGMs, making it impossible to make confrontations with the cisgender, heterosexual population. It should be noted, however, that the recent literature showed no significant differences in terms of knowledge, risk awareness and vaccination attitude between SGMs and cisgender, heterosexual subjects as far as HPV was concerned [29]. Moreover, this choice allowed us to include items pertaining solely to SGMs, such as disclosure to healthcare providers.

Lastly, relying on the social media of SGM=health-dedicated associations for the questionnaire’s dissemination might have resulted in a selection bias, as already mentioned. Nevertheless, we thought that this channel had the potential to minimize the risk of influencing the participants’ answers; further, considering the vast user base of social media, the bias was deemed tolerable.

As a future perspective, new questionnaire-based studies could use more diverse sampling settings, such as SGM health clinics, community centres and SGM-dedicated events (e.g., Pride events), and expand the sexual habit investigation to individuals’ whole lifespan. Other vaccines, or vaccine hesitancy in general, could also be taken into consideration by accessing more numerous sample populations.

## 5. Conclusions

Participants of this study showed a good level of knowledge and awareness regarding HPV infection and HPV-related diseases. They also had a generally positive attitude towards vaccination. A significant number of hesitant subjects wanted to receive more accurate information regarding HPV vaccination, highlighting the need to improve communication and information efforts. Apart from SGM-dedicated information campaigns relying on a coordinated effort of healthcare systems and dedicated organizations, healthcare providers should be trained to communicate effectively to patients belonging to these groups, as well as to provide them with personalized information.

The vaccine’s cost was once again identified as a barrier to vaccination, suggesting the need to implement vaccination offer strategies, including co-payments and the extension of free vaccination to highly vulnerable people outside of the currently targeted age ranges (e.g., SGM adults over 26). Finally, a welcoming environment should be granted to all subjects accessing healthcare, in order to encourage disclosure of sexual orientation and gender identity and make the identification of high-risk subjects by healthcare providers easier. By doing so, healthcare systems would also meet the current recommendations of the Cervical Cancer Elimination Initiative [53].

## Figures and Tables

**Table 1:** Correctness scores for each item of the questionnaire’s knowledge and awareness sections.

Item	Mean Score ± Standard Deviation
*Knowledge*	
HPV is only acquired via penetrative sexual contact.	5.97 ± 1.78
HPV may cause anogenital warts and condylomas.	6.26 ± 1.35
HPV may cause cervical cancer.	6.43 ± 1.25
HPV may cause penile and anal cancer.	5.31 ± 1.85
HPV does not cause oropharyngeal cancer.	4.56 ± 2.32
Using a condom during sexual intercourse lowers the risk of HPV transmission.	6.09 ± 1.37
Having many sexual partners increases the risk of getting infected with HPV.	5.67 ± 1.89
HPV infection can be diagnosed early via the PAP-test.	5.69 ± 1.81
*Awareness*	
I do not think I am at risk of getting infected with HPV.	4.66 ± 2.36
HPV vaccination is effective in preventing the infection.	6.04 ± 1.50
HPV vaccination is exclusive for subjects with female biological sex.	5.90 ± 1.77
A person vaccinated against HPV does not have any residual risk of infection nor cancer for life.	5.45 ± 1.66
HPV vaccination may cure an existing HPV infection.	5.63 ± 1.68
HPV vaccination is not safe for human health.	6.53 ± 1.28

**Table 2:** Study population characteristics.

Qualitative Variables						N.	Percentage
Gender identity	Male					90	50.85%
	Female					70	39.55%
	Non-binary					17	9.60%
Transgender			Yes			16	9.04%
			Transitioning/transitioned		Yes	9	56.25%
					No	7	43.75%
Gender reaffirming hysterectomy				Yes		/	0.00%
Education level				Middle school or lower		9	5.08%
				High school		62	35.03%
				University or higher		106	59.89%
Sexual partners over the last 12 months				≥1 partner with male genitals		117	66.10%
				≥1 partner with female genitals		52	29.38%
				No partners		29	16.38%
Stable sexual partner during the last 12 months						91	51.41%
Regular STD screening						77	43.50%
STD recommended by a healthcare provider						24	13.56%
Disclosure to a healthcare provider						62	35.03%
History of HIV seropositivity						8	4.52%
History of HPV-related lesions						35	19.77%
Knows about HPV						173	97.74%
Knows about the HPV vaccination						157	88.70%
Attitude towards HPV vaccination		Hesitant/refuses				56	31.64%
		Vaccinated				79	44.63%
		Willing to vaccinate				42	23.73%
**Quantitative variables**						**Mean ± Standard Deviation**	
Age						30.14 ± 7.87 years	
School years						15.74 years ± 2.96 years	
N. of sexual partners over the last 12 months			With male genitals			8.32 ± 15.66 (median: 3; interquartile range: 1–6)	
			With female genitals			1.71 ± 1.23 (median: 1; interquartile range: 1–2)	
			Overall			6.00 ± 13.24 (median: 1; interquartile range 1–5)	
Knowledge score						45.98 ± 6.14	
Awareness score						34.21 ± 4.62	

## Data Availability

Data are unavailable due to privacy restrictions.

## Appendix A

### Appendix A.1. Full Text of the Study Questionnaire. E-mails of Involved Staff Were Omitted for Privacy’s Sake

Good morning, and thank you for accepting to participate to our study. We are investigating knowledge about HPV and awareness about the risks related to HPV infection in people belonging to sex and gender minorities. Results will help us plan near future’s healthcare policies and information and communication programs dedicated to specific population groups.

We ask that you answer the following questionnaire, which will take a few minutes. The questionnaire is anonymous. Once you fill it in, you may write your e-mail address if you wish to receive a copy of the results.

Collected information will be managed manually and digitally by staff from the Interdisciplinary Department of Medicine, Aldo Moro University of Bari, Piazza G. Cesare, 11–70124–Bari. Your data will neither be communicated to nor shared with third parties, and will be studied exclusively in an aggregated form for research reasons only.

Your cooperation will be important in order to obtain significant results from this study.

Thank you

[E-mail addresses of responsible staff]

Aldo Moro Bari University

☐ I have understood and I consent to participate anonymously to the study.

☐ I do not consent to participate to the study (the questionnaire will be closed and discarded).

(1) Personal information

• Age in years: ____
• Please select your gender identity:
  ☐ Male    ☐ Female    ☐ Non-binary
• Please state the Region or Autonomous Province you live in:
  ______________________
• Do you identify as a trans person?
  ☐ Yes    ☐ No
• If your answer was yes, have you undergone/are you undergoing a transition process?
  ☐ Yes    ☐ No
• Have you ever undergone a gender reaffirmation hysterectomy (surgical removal of the uterus)?
  ☐ Yes    ☐ No
• Please select your education level:
  ☐ Elementary school
  ☐ Middle school
  ☐ High school
  ☐ University degree of higher education

(2) Sexual anamnesis

• Select the gender of people you had sexual relationships with (one or more):
  ☐ Male
  ☐ Female
  ☐ Other gender identity
  ☐ I have never had any sexual partner
• How many sexual partners with male genitals did you have over the last 12 months? ____
• How many sexual partners with female genitals did you have over the last 12 months? ____
• Have you been having a stable sexual partner, over the last 12 months?
  ☐ Yes    ☐ No
• Do you undergo regular sexually transmitted disease (STD) screening?
  ☐ Yes    ☐ No
• Has a healthcare provider ever suggested you underwent STD screening?
  ☐ Yes    ☐ No
• Have you ever disclosed you gender identity/sexual orientation to a healthcare provider? ☐ Yes     ☐ No
• Have you ever been diagnosed with HIV seropositivity?
  ☐ Yes    ☐ No
• Have you ever been diagnosed with Human Papillomavirus-related lesions (condylomas, anogenital warts, cancerous or precancerous lesions)?
  ☐ Yes    ☐ No

(3) Knowledge

• Have you ever heard of Human Papillomavirus (HPV)?
  ☐ Yes     ☐ No
• If so, who/where from? (Select all that applies)
  ☐ TV/Magazines
  ☐ Friends, relatives, partners
  ☐ Healthcare providers
  ☐ Internet/social media
  ☐ LGBTQ+ associations
  ☐ Others (specify) _______________
• Provide a score from 1 to 7 for each of the following sentences, with 1 = “I totally disagree” and 7 = “I totally agree”
  HPV is only acquired via penetrative sexual contact
  ☐ 1    ☐ 2     ☐ 3     ☐ 4    ☐ 5    ☐ 6     ☐ 7
  HPV may cause anogenital warts and condylomas
  ☐ 1    ☐ 2     ☐ 3     ☐ 4    ☐ 5    ☐ 6     ☐ 7
  HPV may cause cervical cancer
  ☐ 1    ☐ 2     ☐ 3     ☐ 4    ☐ 5    ☐ 6     ☐ 7
  HPV may cause penile and anal cancer
  ☐ 1    ☐ 2     ☐ 3     ☐ 4    ☐ 5    ☐ 6     ☐ 7
  HPV does not cause oropharyngeal cancer
  ☐ 1    ☐ 2     ☐ 3     ☐ 4    ☐ 5    ☐ 6     ☐ 7
  Using a condom during sexual intercourse decreases the risk of HPV transmission
  ☐ 1    ☐ 2     ☐ 3     ☐ 4    ☐ 5    ☐ 6     ☐ 7
  Having many sexual partners increases the risk of getting infected with HPV
  ☐ 1    ☐ 2     ☐ 3     ☐ 4    ☐ 5    ☐ 6     ☐ 7
  HPV infection can be diagnosed early via the PAP-test
  ☐ 1    ☐ 2     ☐ 3     ☐ 4    ☐ 5    ☐ 6     ☐ 7
• Have you ever heard of the HPV vaccination?
  ☐ Yes    ☐ No
• If yes, who/where from? (Select all that applies)
  ☐ TV/Magazines
  ☐ Friends, relatives, partners
  ☐ Healthcare providers
  ☐ Internet/social media
  ☐ LGBTQ+ associations
  ☐ Others (specify) _______________

(4) Awareness

• Provide a score from 1 to 7 for each of the following sentences, with 1 = “I totally disagree” and 7 = “I totally agree”
  I do not think I am at risk of getting infected with HPV
  ☐ 1    ☐ 2    ☐ 3    ☐ 4    ☐ 5    ☐ 6    ☐ 7
  HPV vaccination is effective in preventing the infection
  ☐ 1    ☐ 2    ☐ 3    ☐ 4    ☐ 5    ☐ 6    ☐ 7
  HPV vaccination is exclusive for subjects with female biological sex
  ☐ 1    ☐ 2    ☐ 3    ☐ 4    ☐ 5    ☐ 6    ☐ 7
  A person vaccinated against HPV does not have any residual risk of infection nor cancer for life
  ☐ 1    ☐ 2    ☐ 3    ☐ 4    ☐ 5    ☐ 6    ☐ 7
  HPV vaccination may cure an existing HPV infection
  ☐ 1    ☐ 2    ☐ 3    ☐ 4    ☐ 5    ☐ 6    ☐ 7
  HPV vaccination is not safe for human health
  ☐ 1    ☐ 2    ☐ 3    ☐ 4    ☐ 5    ☐ 6    ☐ 7

(5) Attitude

• Do you intend to get vaccinated against HPV?
  ☐ I already am ☐ Yes    ☐ No
• If your answer was no, why? (Select all that applies)
  ☐ I did not know a vaccine existed
  ☐ Vaccination costs too much
  ☐ I am afraid of adverse events
  ☐ I have already been diagnosed with a HPV infection
  ☐ I do not think I need it
  ☐ Reaching vaccination services is troublesome for me
  ☐ I am afraid of others’ judgement
  ☐ I do not believe the vaccine works
  ☐ I do not trust pharmaceutical companies
  ☐ My physician never recommended it to me
  ☐ Others (specify)________________________________
• If no, what would change your mind?
  ☐ Receiving more information by a healthcare provider
  ☐ A Ministry of Health recommendation for HPV vaccination in LGBTQ+ people
  ☐ Being actively called to vaccination
  ☐ I would not change my mind right now
  ☐ Others (specify)________________________________
• Would you like to receive more information about HPV and its vaccination?
  ☐ Yes    ☐ No

Thank you for your time. The study results will be shared via papers in international journals. If you wish to receive the results, please write you e-mail address: ____________________________________
